# Lack of RsmA-Mediated Control Results in Constant Hypervirulence, Cell Elongation, and Hyperflagellation in *Pectobacterium wasabiae*

**DOI:** 10.1371/journal.pone.0054248

**Published:** 2013-01-23

**Authors:** Viia Kõiv, Liis Andresen, Martin Broberg, Jekaterina Frolova, Panu Somervuo, Petri Auvinen, Minna Pirhonen, Tanel Tenson, Andres Mäe

**Affiliations:** 1 University of Tartu, Institute of Molecular and CellBiology, Tartu, Estonia; 2 University of Helsinki, Department of Biosciences, Division of Genetics, Helsinki, Finland; 3 University of Helsinki, Institute of Biotechnology, DNA Sequencing and GenomicsLaboratory, Helsinki, Finland; 4 University of Helsinki, Department of AgriculturalSciences, Helsinki, Finland; 5 University of Tartu, Institute of Technology, Tartu, Estonia; University of Helsinki, Finland

## Abstract

The posttranscriptional regulator RsmA controls the production of plant cell wall degrading enzymes (PCWDE) and cell motility in the *Pectobacterium* genus of plant pathogens. In this study the physiological role of gene regulation by RsmA is under investigation. Disruption of *rsmA* gene of the *Pectobacterium wasabiae* strain, SCC3193 resulted in 3-fold decrease in growth rate and increased virulence. The comparison of mRNA levels of the *rsmA^−^* mutant and wild-type using a genome-wide microarray showed, that genes responsible for successful infection, i.e. virulence factors, motility, butanediol fermentation, various secretion systems *etc.* were up-regulated in the *rsmA^−^* strain. The *rsmA^−^* strain exhibited a higher propensity to swarm and produce PCWDE compared to the wild-type strain. Virulence experiments in potato tubers demonstrated that in spite of its more efficient tissue maceration, the *rsmA^−^* strain's ability to survive within the host is reduced and the infection site is taken over by resident bacteria. Taken together, in the absence of RsmA, cells revert to a constitutively infective phenotype characterized by expression of virulence factors and swarming. We hypothesize that lack of control over these costly energetic processes results in decreased growth rate and fitness. In addition, our findings suggest a relationship between swarming and virulence in plant pathogens.

## Introduction

**Figure 1:**
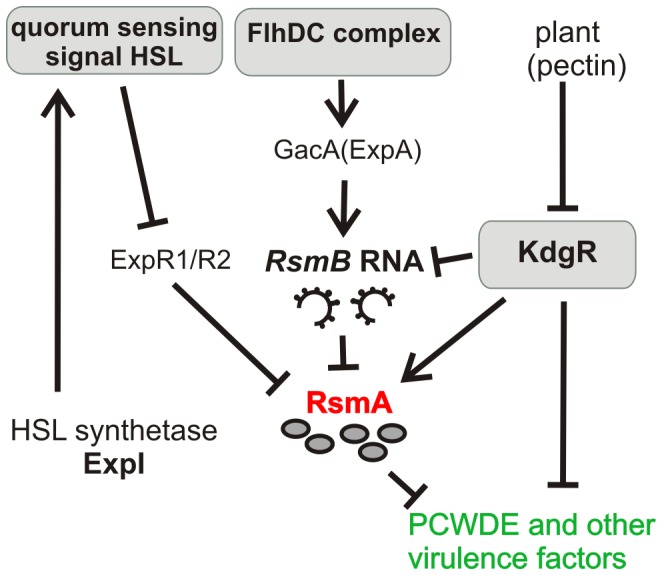
RsmA has a central role in regulating virulence factor synthesis in *Pectobacteria*. Arrowheads indicate positive regulatory effects and barred lines indicate negative effects.

Members of the *Pectobacterium* genus are plant pathogens responsible for causing soft rot in numerous types of plants, including economically important carrot and potato crops. The main factors of pathogenicity involve bacterial motility and production of plant cell wall degrading enzymes (PCWDE). The latter include pectinases, cellulases, and proteases, and these are produced in large amounts following contact with a host plant [1]. To avoid a counterattack by a host, a pathogen attack must be fast and intense, thereby making it energetically expensive. Therefore, the expression of virulence factors is tightly controlled by environmental factors via a cellular regulatory network. In the genus *Pectobacterium*, there are three major, partly overlapping regulatory systems (Fig. 1). One involves the repressor protein, KdgR, which suppresses the production of PCWDE without induction by host-related substances [2]. Second pathway involves quorum sensing that suppresses production of virulence factors before high population densities are reached within a plant host [3], [4]. In the third pathway, the flagellar master regulator, FlhDC, promotes the production of both flagella and PCWDE. Thus, in *Pectobacterium* motility and production of virulence factors are coupled [5], [6].

The effect of quorum sensing on virulence factor production is mediated through the posttranscriptional regulator, RsmA [7]. RsmA binds to a target mRNA to down-regulate its translation and promote its degradation [8], [9], Andresen, L, unpublished data. FlhDC contributes to the control of virulence factor production through GacA (ExpA), a positive regulator of the small regulatory RNA, *RsmB* [6]. *RsmB* binds RsmA, thereby releasing mRNA and neutralizing the negative effect of RsmA on mRNA translation. Hence, both quorum sensing and FlhDC contribute to RsmA-mediated posttranscriptional repression of virulence factors synthesis. In addition, although KdgR directly controls extracellular enzyme expression [10], Hyytiäinen *et al.* have demonstrated that KdgR controls also expression of the *rsmB* and *rsmA* genes [11]. Consequently, the final regulatory step of virulence factor synthesis involves the posttranscriptional repressor RsmA (Fig. 1).

RsmA is a homolog of the RNA binding protein, CsrA, which was first described in *E. coli* as a regulator of glycogen biosynthesis [12]. In addition to controlling glycogen biosynthesis, CsrA and its homologs have widespread regulatory functions in bacteria, including roles in motility [13], central carbon metabolism [14] in *E. coli*, secondary metabolism, and numerous functions involving interactions between *Pseudomonas fluorescens* and plant hosts [15]–[17]. Correspondingly, entries in the protein families database, Pfam, indicate that homologues of CsrA/RsmA are present in a nearly half of bacterial species [18]. In some enterobacterial species, the *rsmA* gene has been shown to be essential since its knock-out strain is not viable [19], [20]. However, depending on the growth conditions, inactivation of *csrA* in *E. coli* has been achieved [21]. Moreover, in *Serratia marcescens* and *Pectobacterium carotovorum*, *rsmA*-defective strains have also been reported [22], [23].

Here, we investigate the global regulatory role of RsmA in *Pectobacterium wasabiae* SCC3193 (*Pw*). Both microarray analysis and physiological characterizations of the *rsmA^−^* mutant are reported. Our analysis reveals that RsmA is a key regulator involved in adaptation of *Pw* for infection/colonization of host plant.

## Materials and Methods

### Bacterial strains and plasmids

The *Pectobacterium wasabiae* wild-type strain, SCC3193 (previously identified as *Pectobacterium carotovorum* subsp. *carotovorum*), and its *rsmB*-defective mutant have been described previously [24]–[26]. To construct a *Pw rsmA*-deficient strain, the *rsmA* sequence from the genomic DNA of SCC3193 was PCR amplified using primers, RAP17 (5′-TCTTTCAAGGAGCAAAGAATGC-3′) and RAP18 (5′-CGCGAACACGAGACGCATTG-3′), and was subsequently cloned into the pUTmini-Tn5Km vector [27]. In the resulting construct, the *rsmA* gene was inactivated by cloning the *cat* gene (Cm^R^) into the *Bs*tEII restriction site. This vector construct was then used as a template to amplify *rsmA::Cm* fragment (using primers RAP17 and RAP18), which was used to generate a *rsmA*-defective strain according to the method described by Datsenko and Wanner [28]. The resulting strain contained a chromosomal insertion of the chloramphenicol resistance gene 64 nucleotides downstream of the translational start codon of the *rsmA* coding sequence.

PCR-amplified *rsmA* (using primers RAP17 and RAP18) and *rsmB* [using primers rsmB2 (5′-AATACATCTTATTACTTAAG-3′) and AE16AEPA (5′-AGCCAAGTGTGACTGACAGCATTTTT-3′)] genes were cloned into the Bluescript SK plasmid (Stratagene) and used to generate respective overexpression constructs.

The pLATSprtW construct was used to measure expression of *prtW*. This construct contained the *prtW* gene promoter and twelve amino acids of coding sequence that were PCR amplified using primers, prtWpA (5′-TGCAGATACTGAGGTATCGT-3′) and PROM8 (5′-AAGCTTACCCAGTGCGTCGTT-3′). The amplified fragment was cloned in frame with the *gusA* reporter gene in the low-copy vector, pMW119 (Eurogentec).

### Virulence assay in tobacco seedlings

Virulence of the *Pw* wild-type and mutant strains was tested in 3-to-4-week-old tobacco seedlings (*Nicotiana tabacum* var. Samsun), grown in 24-well tissue culture plates in Murashige-Skoog medium. Seedlings were surface inoculated by pipetting bacterial solution (10^8^ bacterial cells) onto plants without wounding. The inoculated plants were incubated in a plant growth chamber with high humidity. The development of disease symptoms (i.e., tissue maceration) was monitored for 24–72 h after inoculation.

### Gene transcription level profiling

Total RNA was isolated from wild-type and *rsmA*-defective strains grown on M9 minimal plates containing 10% potato tuber extract using Trizol reagent according to the manufacturer's protocol (Invitrogen). To eliminate any traces of genomic DNA prior to RNA amplification, an additional DNase I treatment was performed according to the supplier's protocol (Fermentas).

Extracted RNA from samples was checked for sufficient purity using NanoDrop and an Agilent bioanalyzer (containing a RNA Nano 6000 chip) prior to being treated with a MICROBexpress kit (Ambion) to remove 16S and 23S rRNAs. According to the manufacturer, this process significantly increases the sensitivity of downstream applications such as microarrays. Purified mRNA from each sample was checked for sufficiently low rRNA contamination using a bioanalyzer, and then was treated with a MessageAmp™ II-Bacteria RNA Amplification kit (Applied Biosystems) according to the manufacturer's instructions. This kit was supplemented with 5-(3-aminoallyl)-UTP, ultimately producing antisense RNAs (aRNAs) which were subsequently labeled with fluorescent dyes (based on binding to the aminoallyl-UTP). The three different dyes used were: Alexa488 (Invitrogen), Cy3, and HyPer5 (Amersham). aRNA (5 µg)for each biological replicate was dried in a vacuum centrifuge, then resuspended in 7 µl sterile MQ water and 9 µl Na_2_CO_3_ buffer (pH 9.3). Fluorescent dye (4 µl) was then added, and a different dye was incubated with each biological replicate. Samples were subsequently incubated at RT in darkness for 1 h, then for an additional 15 min following the addition of 4M hydroxylamine (4.5 µl/sample). Samples were purified using a PureLink PCR purification kit (Invitrogen), then hybridized on an Agilent microarray for 17 h in a G2545 Agilent hybridization oven, according to the manufacturer's instructions (One-Color Microarray-Based Gene Expression Analysis Low Input Quick Amp Labeling, v6.5). The Agilent 8*15k custom microarray contains 60mer probes covering a total 4571 ORFs.

The microarray slide was scanned using a Genepix 4110AL scanner (Axon Instruments) and analyzed with Genepix pro 6.1. Data were also analyzed using Bioconductor, a limma software package for the R programming language. Normalization was achieved using quantile normalization, empirical Bayesian variance shrinkage was used for statistical analysis, and data were combined from all probes for each gene. A false discovery rate (fdr) value of 0.05 was used as the cut-off for significance in statistical analyses.

### Extracellular enzyme assays

For the quantitative analysis of extracellular pectatelyases, polygalacturonase, and proteases, strains were grown in M9 minimal media [29] supplemented with appropriate trace elements [30] and a carbon source (0.2% glucose or 0.2% polygalacturonic acid). Extracellular enzyme activities were measured in cell-free supernatant samples according to previously published protocols [31], [32]. To perform semi-quantitative agarose plate assays of extracellular protease and polygalacturonase production, cells were spotted on respective indicator plates described by Chatterjee and coworkers [23], with the exception that a milk-containing agar plate additionally contained 0.05% polygalacturonic acid as an inducer for proteases. Plates were incubated at 30°C and observed 24 and 72 h after incubation (for pectinolytic enzymes and proteases, respectively). Enzyme activity was evaluated according to the size of the halo around each colony, which is proportional to the amount of secreted enzyme.

### Analysis of prtW expression

Strains containing the *prtW::gusA* translational fusion (pLATSprtW) were grown in M9 minimal medium supplemented with 10% celery extract. GusA activity was measured in the exponential and early stationary phases of growth using 4-methylumbelliferyl *β*-d-glucuronide (DUCHEFA Biochemie) as a substrate, according to Andresen *et al.* [5].

### Identification of extracellular proteins

Cell-free supernatants were collected from cultures grown in minimal glucose and PGA medium, and these samples were concentrated 20 times by acetone precipitation. Proteins were separated by SDS-PAGE (11%) and protein bands were visualized using Comassie Blue R-250 staining. Proteins were cleaved with trypsin and LysC proteases, analyzed by mass spectrometry (LTQ Orbitrap), and identified with searches against the SwissProt database using Mascot software.

### Motility tests

The swimming phenotype of *Pw* strains were evaluated by stabbing fresh cultures with sterile toothpicks and then inserting the toothpicks into 0.25% soft agar plates containing M9 minimal medium with trace elements and 0.4% polygalacturonic acid as a carbon source.

Swarming motility was assayed by spotting each strain onto minimal plates containing 0.4% agar and 10% potato tuber extract.

Both swimming and swarming motility phenotypes for each strain were evaluated visually by observing the migration of cells away from the inoculation site.

### Flagella staining

Macerated potato tuber tissues (∼0.2 g each) were gently soaked in 1 ml distilled water, cut into small pieces without any agitation, and placed in tubes. Samples were left to stand for ∼15 min until the pieces of potato precipitated. The upper bacterial suspension was then removed (∼500 µl) to a new Eppendorf tube and centrifuged for 3 min at 100 g. The upper aqueous phase was removed and the cells were gently resuspended in an appropriate volume of distilled water. This bacterial solution (10 µl) was immediately pipeted onto a microscope slide and left to dry very slowly under a cover before staining of flagella was performed according to West *et al* [33].

### Glycogen accumulation

The glycogen content of each strain was evaluated on solid Kornberg medium (1.1% K_2_HPO_4_, 0.85% KH_2_PO_4_, 0.6% yeast extract, 1% glucose) agar plates. Strains were grown on these plates for 24 h at 30°C then exposed to iodine vapor for 10 min to assess glycogen accumulation.

### Characterization of butanediol fermentation products

Potato tubers were inoculated with 5×10^6^ bacterial cells suspended in 10 mM MgSO_4_. After 24 and 48 h at 30°C, tubers were cut in half and macerated tissue present was removed from the inoculation site into Eppendorf tubes. Following centrifugation at 15 700 g for 10 min, supernatants were analyzed using the Voges-Proskauer test [34]. A red color indicated the presence of acetoin, and its accumulation was quantified at OD530 nm.

### Virulence assay in potato tubers

Surface-sterilized potato tubers (cv. Ants) were stabbed with a sterile pipette tip and solutions of wild-type or *rsmA^−^* cells (5×10^6^) in 10 mM MgSO_4_ were transferred into the wound. Negative controls were inoculated with sterile 10 mM MgSO_4_. Tubers were kept at 30°C under plastic to maintain moist conditions. Virulence was estimated by weighing the rotted tissue present in tubers 20 and 40 h after inoculation.

To estimate the number of bacteria present in the infection sites, *ca* 2.5 g of tissue surrounding the infection site was resected, weighed, and homogenized in sterile 0.9% NaCl. Dilutions for CFU counting were plated on LB agar plates, except for *rsmA^−^* cells which were plated on LB plates containing Cm. Colonies were counted after plates were incubated for 24 or 72 h at 30°C for wild-type and *rsmA^−^* strains, respectively. To distinguish between *Pw* and other species of bacteria inhabiting the tubers, colonies were tested for the production of PCWDE on indicator plates.

## Results

### Construction of the rsmA knockout

To generate a knockout mutant of *rsmA* in the *Pw*, a Cm resistant marker was inserted into the *rsmA* gene 64 nucleotides downstream of the translational start codon. The resulting *rsmA^−^* strain was observed to have a slower growth rate than the wild-type *Pw*, independent of the medium used. To examine the physiological parameters of *rsmA* ^−^ growth, liquid minimal medium containing a sole carbon source, 0.2% glucose or 0.2% polygalacturonic acid (PGA) was used. PGA mimics a plant component for induction of PCWDE. The *rsmA^−^* was compared to both wild-type and *rsmB* ^−^ strains. The generation times for wild-type, *rsmB* ^−^, and *rsmA* ^−^ strains in 0.2% glucose were 73, 75, and 240 min, respectively. In 0.2% PGA, the generation times were 85, 88, and 230 min, respectively. Accordingly, the *rsmA* ^−^ strain grows 2.5–3 times more slowly than wild-type or *rsmB^−^* strains.

Next, the virulence of the *rsmA* ^−^ mutant was compared with wild-type and *rsmB^−^* strains in 3-to-4-week-old tobacco seedlings (Fig. S1). Three days post inoculation, there were no disease symptoms observed in plants infected with the *rsmB^−^* mutant. In contrast, 63% of *rsmA^−^*-infected plants, and 22% of plants infected with the wild-type strain, were completely macerated. Based on these results, the *rsmA* ^−^ strain appears to have increased virulence despite exhibiting a reduced growth rate.

### RNA microarray analysis

To elucidate the mechanism(s) mediating the increase in tissue maceration associated with the *rsmA* ^−^ mutant, global transcript level analysis was performed for *Pw*. For these studies, a DNA microarray containing 4571 open reading frames (ORFs) of *Pw* was used. Total RNAs were extracted from wild-type *Pw* and the *rsmA^−^* strain that were grown on 1.5% agar containing solid minimal medium supplemented with 10% potato extract to mimic *in planta* growth conditions. Based on the differences in growth, samples were collected from the *rsmA^−^* strain 40 and 48 h post inoculation, while wild-type samples were collected after 24 h. RNA pools for these samples were then hybridized to microarrays and the gene transcript level profiles obtained were compared.

Although differences in the mRNA expression levels between wild-type and *rsmA* ^−^ strains were not large, 39% of the ORFs analyzed (i.e., 1800 ORFs) showed statistically significant differences between the two strains (p≤0.05). In addition, the number of ORFs with differences between the *rsmA^−^* strain and wild-type was considerably higher at the 40 h time point than at the 48 h time point (1631 *vs.* 565, respectively). The number of ORFs exhibiting differences at both time points was 396. In all cases, approximately half of the differentially expressed ORFs were transcribed at higher levels in the *rsmA^−^* strain than in the wild-type strain (available in NCBI GEO database, seires accession: GSE40333; ID: 200040333).

**Figure 2:**
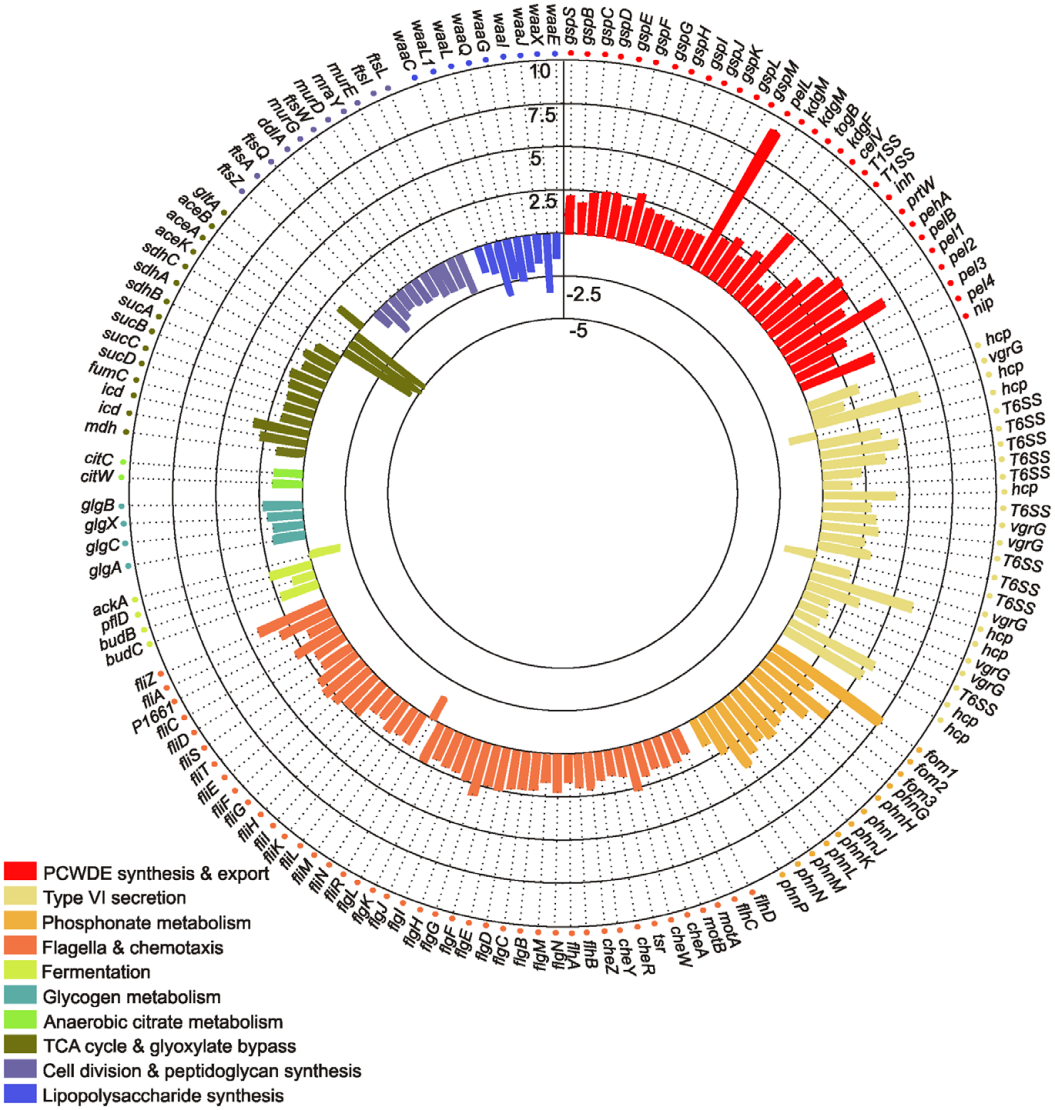
In *Pectobacteria rsmA* expression level affects transcriptome. A global mRNA profile of the *rsmA* ^−^ of *Pw* was compared with its parental strain. The results were compared with a previous study of the *expI* ^−^ strain of *Pa* [3]. In the *expI* ^−^ strain the RsmA level is increased [7]. The genes included in the figure are those which passed two criteria: firstly, the expression level changes were in the opposite direction in response to *rsmA* inactivation as compared to *rsmA* up-regulation (*expI^−^* strain); secondly, the genes have been associated with virulence. The scale bar(top of figure) represents the fold change in mRNA levels between the wild-type and the *rsmA^−^ Pw* strain, with positive values representing up-regulation and negative values representing down-regulation in the *rsmA^−^* strain.

To analyze the universal role of *rsmA* in *Pectobacteria*, the results of the *Pw* microarray were compared with previously reported results from a microarray analyzing mRNA level profiles of wild-type *P. atrosepticum (Pa)* versus an *expI^−^* strain [3]. The inactivation of *expI* leads to increase in RsmA level (Fig. 1) [7]. Although the conditions in which the cells were grown prior to RNA extraction were different (plates containing potato extract for *Pw vs.* potato plant for *Pa*), there were several genes with mRNA levels showing trends in opposite direction in the two experiments, as would be expected for the opposite *rsmA* expression levels (Fig. 2). In addition to known virulence factors, PCWDE and flagella, that were up regulated in *rsmA^−^* strain and down regulated in *expI^−^* strain, there were several groups of genes showing large effects: type VI secretion system, the phosphonate cluster, glycogen metabolism, citrate uptake and degradation, butanediol fermentation and TCA cycle, whereas the genes for cell division, peptidoglycan synthesis and lipopolysaccharide synthesis were down regulated in *rsmA^−^* strain and up regulated in *expI^−^* strain (Fig. 2).

In subsequent experiments, the contribution of RsmA to infection/virulence-linked physiological traits and the general energetic state of the cell are further examined.

### Plant cell wall degrading enzymes (PCWDE)

**Figure 3:**
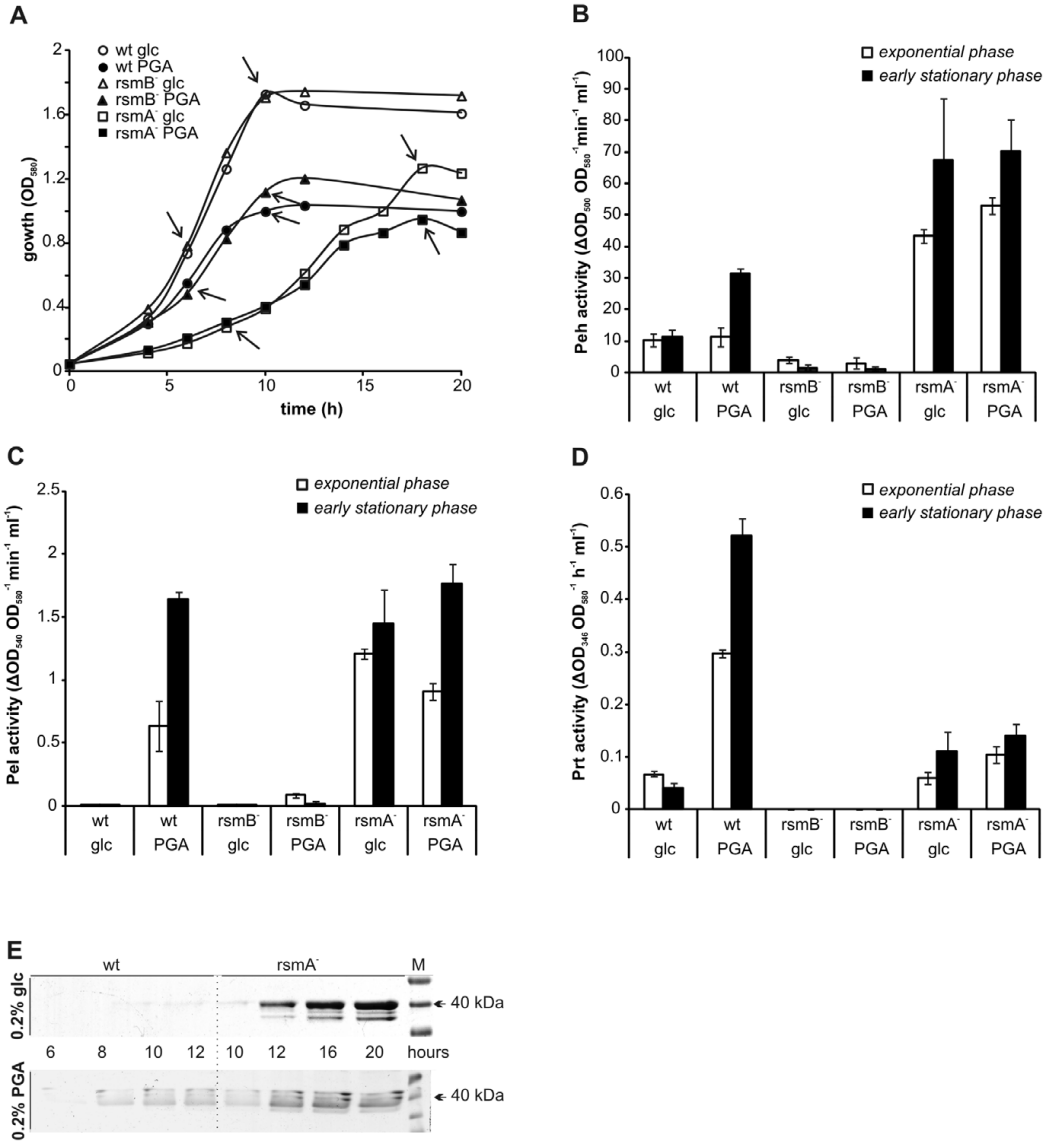
The *rsmA* mutant produces high levels of pectinolytic enzymes which constitute the majority of the proteins that are secreted. Wild-type (wt), *rsmA^−^*, and *rsmB^−^* cells were grown in minimal medium containing either 0.2% glucose (glc) or 0.2% polygalacturonic acid (PGA). Cell-free supernatants were then collected at various timepoints to evaluate protein content. A. The growth curves obtained for each strain and growth condition. The arrows indicate the timepoints used for collecting supernatant samples. B–D. Activity of polygalacturonase (Peh) (B), pectate lyases (Pel) (C), and proteases (Prt) (D) in supernatant samples collected from the exponential phase (white) and the early stationary phase (black) for each strain indicated. The average activity values for three independent experiments are represented. Error bars indicate the standard deviation values. E. Proteins in the wild-type and *rsmA^−^* strain culture supernatants were concentrated20 times by acetone precipitation and separated by SDS PAGE. M indicates molecular weight marker.

Levels of mRNA for PCWDE and genes coding for export systems for PCWDE, were found to be at higher levels in the *rsmA* defective strain than in the wild-type strain. To better understand these microarray data, the *rsmA^−^* strain and the wild-type strain were grown in liquid minimal medium containing either 0.2% glucose or 0.2% PGA as the sole carbon source. The activity of three PCWDE was then assayed throughout their growth curves. These included polygalacturonase (Peh), pectatelyases (Pel), and protease (Prt) (Fig. 3). In glucose medium, the levels of Peh activity were 5–7 times higher in the *rsmA* ^−^ strain than in the wild-type strain. Moreover, in *rsmA^−^* mutants, levels of Peh activity were similar for glucose and PGA samples, while wild-type cells exhibited a clear induction of Peh activity by the plant component PGA. In contrast, levels of Peh activity in the *rsmB* knockout were not considerable.

Pel activity was also higher in the *rsmA^−^* strain compared to the wild-type strain, and induction by PGA was minimal. For the *rsmB* ^−^ strain, Pel activity was only detectable in cells grown with PGA (Fig. 3).

It was also observed that Pel and Peh production started earlier in the *rsmA^−^* strain compared with the wild-type strain (data not shown). A lack of quorum sensing in the *rsmA^−^*strain is a probable explanation for this phenomenon. Transcription of *rsmA* in wild-type *Pw* is under the negative control of the quorum sensing signal, homoserine lactone (HSL) [7]. ExpR1/R2 proteins activate the transcription of *rsmA* until HSL accumulates, and then HSL binds ExpR1/R2 to release the complex from the promoter region of *rsmA* (Fig. 1) [35]. Thus, in the late exponential phase of wild-type *Pw*, the accumulated HSL removes activation of RsmA synthesis that causes induction of Peh and Pel. In the absence of RsmA this regulatory cascade is interrupted and the knockout strain is constantly in state similar to the wild-type strain in the late exponential phase.

**Figure 4:**
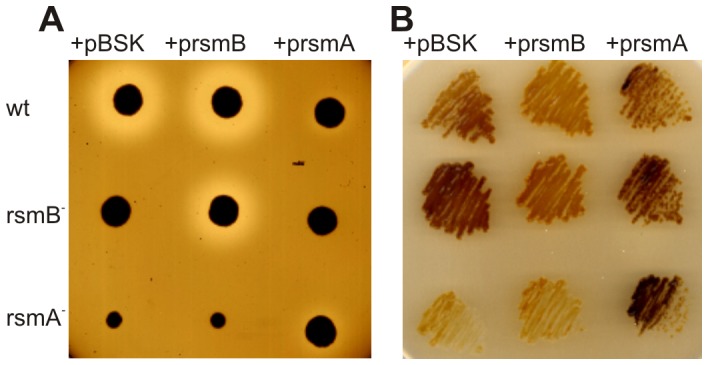
Plasmid expression of *rsmA* or *rsmB* can complement the corresponding knock-out mutants. A. The production of protease (Prt) was assayed using a specific, semi-quantitative indicator plate, where the halo around the inoculation site was proportional to the amount of enzyme produced. Exogenous expression of *rsmA* and *rsmB* (p*rsmA* and p*rsmB*, respectively) was achieved, with pBSK being the vector control. B. To assess glycogen accumulation, cells were grown on Kornberg's medium then exposed to iodine vapors. Darker staining represents higher levels of glycogen. Assays were performed at least three times, and no significant variations were observed.

Based on the microarray data collected, it was hypothesized that the protease activity of the *rsmA^−^* strain would be high. However, although the protease activity of cells grown in glucose was slightly higher in the *rsmA^−^* strain versus the wild-type strain, *rsmA^−^* cells exhibited ∼5-fold lower levels of protease activity in PGA liquid culture than wild-type cells. Moreover, no detectable protease activity was present in *rsmB^−^* cells, indicating that the Rsm system controls protease production. To clarify this apparent discrepancy, we added *rsmA* gene or *rsmB* gene on plasmid to wild type cells. Consistent with microarray data, over-expression of RsmA suppresses the protease production while the small RNA *RsmB* enhances the protease production in wild-type cells (Figs. 3, 4).

To further verify the mRNA level profiling data obtained, the *prtW* regulatory region was fused to the GusA coding region to detect *prtW* expression in wild-type, *rsmA^−^*, and *rsmB^−^* strains. As shown in Figure S2, the expression of GusA was higher in the *rsmA^−^* strain than in the wild-type strain, while in the *rsmB^−^* strain it was lower than in the wild-type strain. These results are consistent with the microarray data, and suggest that expression of *prtW* in the *rsmA* ^−^ strain was down-regulated at the level of protein stability, protein export, or enzymatic activity.

### Extracellular proteins

Although high levels of extracellular Peh and Pel activity were detected in the *rsmA^−^* strain, it was not clear whether higher levels of extracellular protein expression by the *rsmA^−^* strain were limited to PCWDE, or whether a general increase in protein export was involved. To distinguish between these possibilities, culture supernatants of wild-type and *rsmA^−^* strains were concentrated and then separated by SDS-PAGE (Fig. 3E). Using mass spectrometry, the most abundant proteins detected in the *rsmA^−^* growth medium were PehA, Pel1, Pel2, Pel3, and CelV, along with traces of flagellin, FliC, and translation elongation factor, EF-Tu. These results indicate that the increased extracellular protein production associated with the *rsmA* ^−^ strain is specific to PCWDE.

### Motility

**Figure 5:**
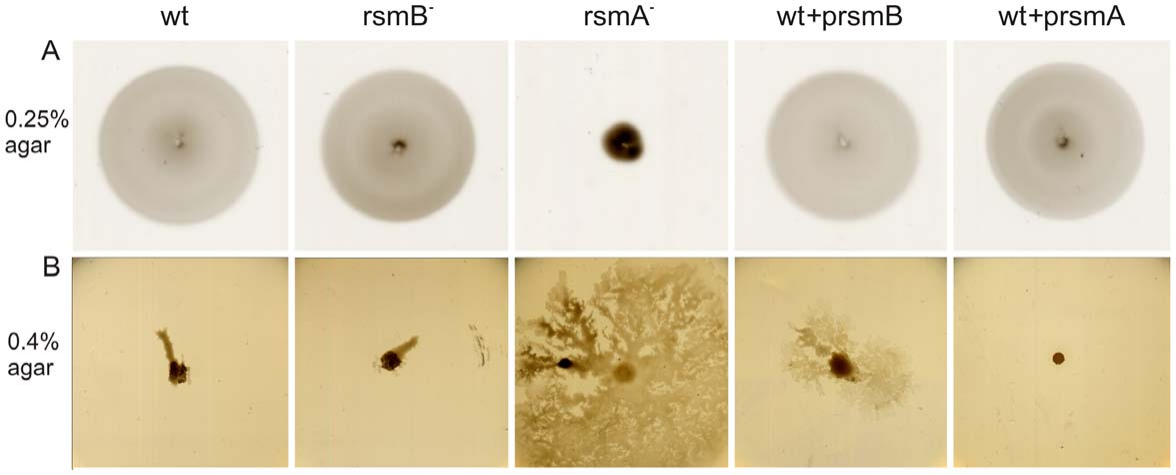
RsmA affects the swimming and swarming motility of *Pw*. A. The swimming ability of wild-type, *rsm* mutants (*rsmA* ^−^ and *rsmB* ^−^), and the respective overexpression strains (wt+prsmA and wt+prsmB) were evaluated based on the migration speed of these cells in 0.25% soft agar plates containing polygalacturonic acid. Cells were imaged 48 h after inoculation. B. The swarming ability of the wild-type, *rsm* mutants (*rsmA* ^−^ and *rsmB* ^−^), and the respective overexpression strains (wt+prsmA and wt+prsmB) were evaluated. The ability of the cells to migrate on the surface of the medium, away from the site of inoculation was examined on 0.4% agar plates containing 10% potato extract. Plates were imaged 24 h after inoculation. Swarming of the wild-type strain was not observed until 72 h after inoculation (not shown). All images are representative of five different experiments performed, and no significant variability was observed between the experiments.

According to the array data, majority of the genes that encode flagella proteins are up-regulated in the *rsmA* ^−^ strain, in accordance with previous studies of the Rsm system [36]. It has also been shown in several bacteria, including *Pectobacteria*, that RsmA/CsrA represses motility [20], [22], [36], [37]. The increased synthesis of flagella proteins and the energy consumption required for active flagella is a huge energetic commitment for a cell. To determine whether increased production of flagella in the *rsmA^−^* strain contributes to the slower growth rate observed, studies of motility, both swimming and swarming, were conducted for *rsmA^−^*, *rsmB^−^*, and wild-type strains (Fig. 5). While the *rsmB^−^* and wild-type strains exhibited similar rates of swimming, *rsmA^−^* cells were unable to spread in the 0.25% agar used for the assay. Moreover, this inability to swim was observed in PGA, glucose and glycerol media but not when 20 amino acids at final concentration of 100 µg/ml each were added to these media (data not shown). In addition, the swimming activity of the three strains was subsequently examined in liquid media using a phase contrast microscope. Cells were grown in 0.2% glucose or 0.2% PGA minimal medium and samples were collected at various points throughout their growth curves. In this assay, all three strains were observed to be motile. This suggests that the knock out strain has functional flagellae. The inability to swim is probably caused by a defect in the signaling that activated swimming *i.e.* chemotaxis.

Swarming motility generally requires an energy-rich, solid medium. However, the conditions specific for inducing swarming can vary for different bacteria [38]. In this study, swarming motility was assayed on 0.4% agar plates with different rich media containing celery extract or potato tuber extract to simulate plant induction. Swarming was only observed on plates containing 10% potato tuber extract. *RsmA^−^* cells were observed to swarm rather quickly across the semi-solid surface, covering it with a hardly visible layer of cells (Fig. 5). In contrast, wild-type cells started to swarm on the third day of incubation (data not shown), while *rsmB^−^* cells remained at the site of inoculation. The increased swarming activity observed for the *rsmA^−^* strain is in accordance with the increased production of flagella detected in the microarray data.

Swarmer cells have been characterized as elongated and hyper-flagellated, and secrete wetting agents to facilitate spreading over semi-solid surfaces [38]–[42]. Swarming bacteria increase the number of flagella per cell in a period of time referred to as the “swarming lag”. As indicated above, the swarming lag for wild-type cells was 3 days, while *rsmA^−^* cells did not exhibit a lag period. Similarly, PCWDE production was found to have a lag period in wild-type cells, yet not in the *rsmA^−^* strain. Taken together, these data suggest that during the lag period in wild-type cells, both swarming and PCWDE production are repressed by RsmA, and this repression can be removed by HSL-mediated quorum sensing.

**Figure 6:**
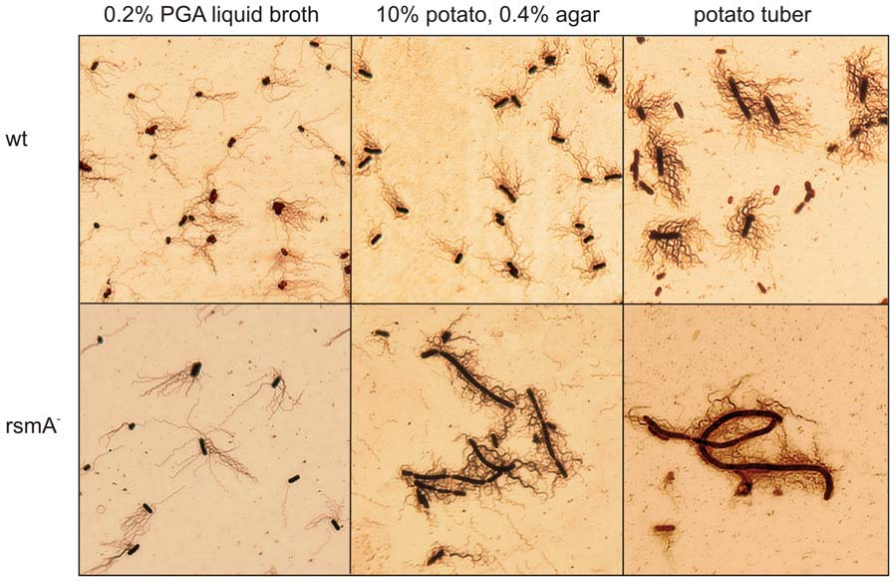
*Pw* cells change their shape and flagellation depending on their growth environment. Wild-type (wt) and *rsmA^−^* strains grown in different media, as indicated, had their flagella stained according to West *et al.* [33]. Representative images of cells grown in the liquid medium (0.2% PGA liquid broth), swarming plate (0.4% agar containing 10% potato extract)and potato tuber are shown. The samples from potato tubers were collected 20 h after inoculation, all the other samples 24 h after inoculation. On the swarming plate the wild-type cells became long and hyperflagellated after 72 h of inoculation (not shown).

Next, silver-stained flagella were examined using a light microscope (Fig. 6). For these assays, cells were suspended in water prior to staining. When *rsmA^−^* cells were collected from a potato extract-containing plate, they were observed to be rather sticky. It was found that the mordant tannin used for thickening flagella was stuck to an unknown substance on the cell surface, resulting in significant distortion of each cell's shape. By performing an additional washing step with water, the cells were able to be separated. In liquid media, *rsmA^−^*, *rsmB^−^*, and wild-type cells of all strains were observed to be rod-shaped and similarly flagellated (Fig. 6). This suggests that the decreased growth rate in liquid culture of the *rsmA^−^*strain is not caused by increased production of flagella. However, when cells were grown for 24 h on plates containing potato extract, wild-type and *rsmB^−^* cells maintained a rod-shape and were “normally” flagellated, while most of the *rsmA^−^* cells were longer and hyper-flagellated in both 0.4% agar (i.e., the swarming plate) and 1.5% agar (i.e., medium from which samples were prepared for microarray analysis). Upon further incubation, the cell morphology of the *rsm* mutants remained unchanged. However, the wild-type cells became longer and more flagellated concurrent with the onset of swarming motility (data not shown).

### TCA cycle, glyoxylate shunt, and glycogen metabolism

**Figure 7:**
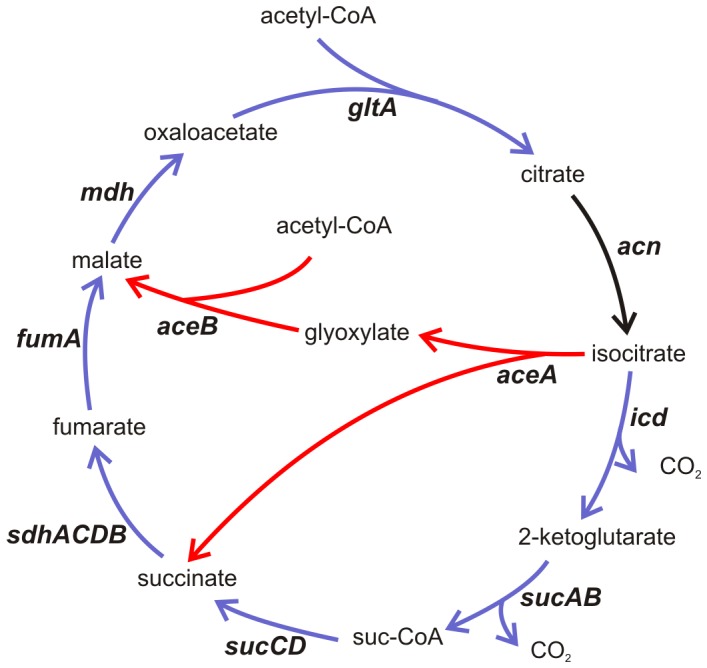
The role of RsmA in regulating the tricarboxylic acid (TCA) cycle and glyoxylate bypass. Blue arrows indicate reactions catalysed by the enzymes corresponding to genes up-regulated in the *rsmA^−^* strain according to microarray data. Red arrows indicate reactions catalysed by the enzymes corresponding to genes down-regulated in the *rsmA^−^* strain. *icd*–isocitrate dehydrogenase; *sucA*–2-ketoglutarate dehydrogenase; *sucCD*–succinyl CoA synthetase; *sdhACDB*–succinate dehydrogenase; *fumA*–fumarase; *mdh*–malate dehydrogenase; *gltA*–citrate synthase; *acn*–aconitase; *aceA*–isocitratelyase; *aceB*–malate synthase.

The reduced growth rate of *rsmA* ^−^ mutants may be linked to a low energy state for these cells that is caused by increased production of virulence factors. The mRNA levels of genes involved in the glyoxylate shunt were found to be down-regulated, and genes for the TCA cycle were up-regulated, in the microarray data obtained (Figs. 2, 7).

The microarray analysis performed detected the up-regulation of genes for glycogen synthesis, including *glgA*, *glgC*, and *glgB*, as well as a gene for glycogen degradation, *glgX*, in the *rsmA^−^* strain (Fig. 2). To confirm these results, glycogen accumulation on solid Kornberg medium was assayed with iodine vapor staining. As shown in Figure 4, the *rsmA^−^* strain had slightly decreased glycogen levels. In *E. coli*, the RsmA homologue, CsrA is a protein originally described as a regulator of carbon metabolism [12]. In *csrA^−^* knock-out, glycogen synthesis is favored and excess glycogen accumulation impairs viability [21]. Thus, in contrast with the *E. coli csrA^−^* mutant, the *Pw rsmA* ^−^ mutant consumes resources rather than saves them, and the increased consumption of glycogen may be linked to the energy deficiency associated with the *rsmA* ^−^ mutant.

### Fermentation

**Figure 8:**
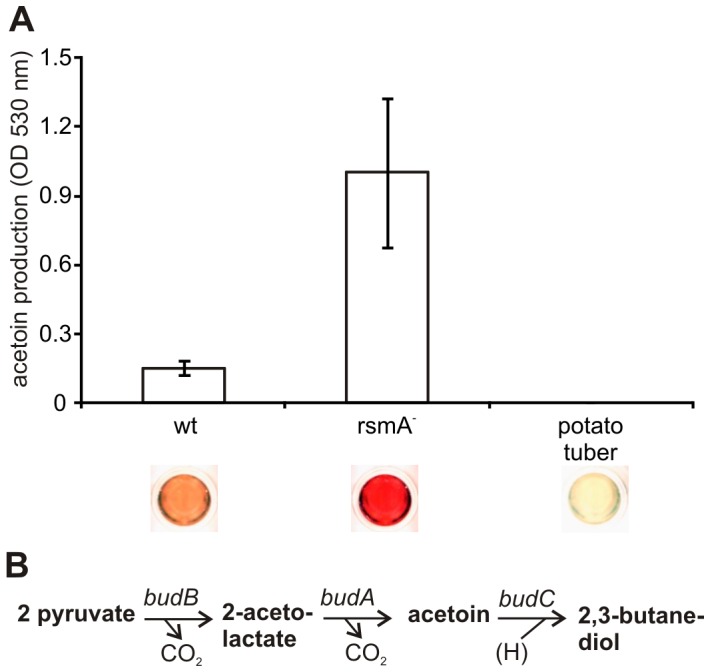
RsmA regulates butanediol fermentation in potato tubers. A. Potato tubers infected with wild-type (wt) or *rsmA^−^* strains were assayed for activity of the butanediol fermentation pathway. Specifically, production of acetoin (an intermediate in the butanediol fermentation pathway) was detected spectrophotometrically (at 530 nm) using the Voges-Proskauer method 24 h after inoculation [34]. The experiment was performed in triplicate and error bars indicate standard deviation values. The colorimetric reaction associated with each result is shown below the graph. B. The butanediol fermentation pathway in *Pectobacteria*. The reactions are catalysed by enzymes coded by *budB* (acetolactate synthase), *budA* (α-acetolactatedecarboxylase), and *budC* (acetoinreductase).

Under the low oxygen conditions of internal plant tissues, plant-associated *Enterobacteriaceae* commonly ferment sugars through the 2,3-butanediol pathway. In the *rsmA^−^* mutant, the genes responsible for 2,3-butanediol production (*i.e.*,*budA*, *budB*, and *budC*), were found to be slightly up-regulated in the microarray data collected (Fig. 2). To confirm these results, levels of acetoin, a predecessor of 2,3-butanediol,were detected in macerated potato tuber tissue infected with wild type or *rsmA^−^* mutant cells. As shown in Figure 8, levels of 2,3-butanediol fermentation were higher in the *rsmA^−^* strain, which accounts for the higher pH of macerated potato tuber tissue collected from the *rsmA^−^* strain compared to tissue infected with the wild-type strain (pH 6 *vs.* pH 5.5, respectively).

The alkalization of growth media by 2,3-butanediol promotes the growth of bacteria that are unable to cope with acidic conditions in the plant. Therefore, the higher pH of potato tissue may contribute to the increased virulence exhibited by the *rsmA^−^* strain (Figure S1).This is in agreement with the previous observations that inactivation of the *bud* genes of phytopathogenic bacteria from the genera, *Dickeya* and *Pectobacterium* reduce the virulence and fitness of these bacteria [43], [44].

### Citrate uptake

**Figure 9:**
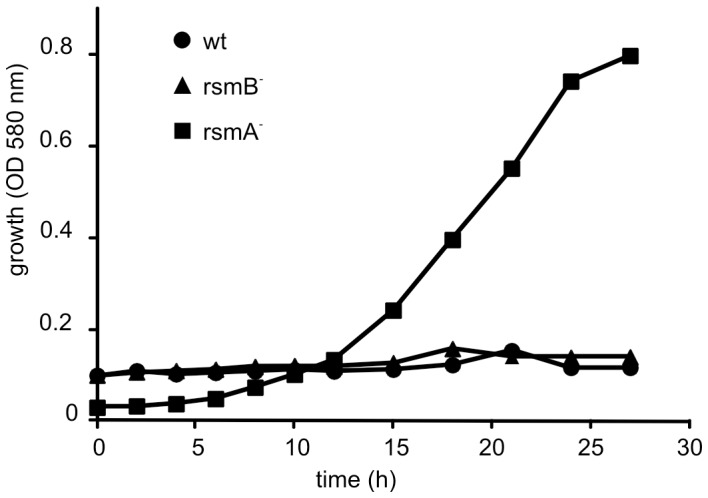
The RsmA mutant is able to grow aerobically on citric acid. Wild-type (wt), *rsmA^−^*, and *rsmB^−^* strains were tested for their ability to grow aerobically in minimal medium containing 0.2% citric acid as the sole carbon source. The absorbance of cultures at 580 nm was recorded up to 27 h post inoculation. Experiments were performed in triplicate.

Citrate is present at high concentrations in the plant apoplast [45], and is transported by a citrate-specific carrier/permeability system into the bacterial cell [46]. Correspondingly, the limiting step for the utilization of extracellular citrate by bacteria is the requirement for specific transporters. In the microarray data collected, increased mRNA levels of the gene cluster for citrate transport (*citW*) and fermentation (citrate lyase *citE*, *citC*) were detected in the *rsmA^−^* mutant (Fig. 2). To confirm these data, wild-type, *rsmB^−^*, and *rsmA^−^* cells were grown in minimal medium supplemented with 0.2% citrate as the sole carbon source. As shown in Figure 9, only *rsmA^−^* cells were able to grow under these conditions. These data suggest that RsmA is involved in regulating the acquisition of different carbon sources during infection.

### Virulence in potato tubers

**Figure 10:**
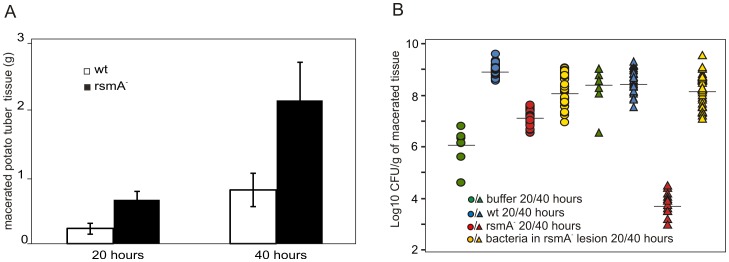
The virulence and survival of wild-type and *rsmA^−^* strains over the course of potato tuber infections. Potato tubers (n = 30) inoculated with 5×10^6^ CFU of wild-type (wt) or *rsmA* ^−^ cells were placed in plastic boxes and incubated at 30°C. A. At 20 h and 40 h post inoculation, macerated tissue was removed from the inoculation site and weighed. Error bars indicate standard deviation values. B. The number of bacteria residing in the infection sites infected with wt, *rsmA* ^−^ cells or buffer was determined by plating dilution series (green, blue and yellow). The number of *rsmA^−^* bacteria present in infection sites was determined using Cm-containing plates to avoid overgrowth by other bacteria inhabiting the infection sites (red). Circles and triangles represent data from the 20 h and 40 h time points assayed, respectively. Horizontal lines represent the median values.

Since potato tuber extracts were shown to induce swarming (Fig. 5), the relationship between swarming and potato tuber infections was investigated. For these studies, potato tubers were infected with wild-type or *rsmA^−^* cells. At time points of 20 and 40 h post-infection, *rsmA^−^* cells were found to produce up to three times more macerated tissue than wild-type cells (Fig. 10A).

Since the generation time of the *rsmA^−^* strain is 3–4 times slower than that of the wild-type strain, the ability of the *rsmA^−^* strain to multiply in potato tubers was studied. For these studies, dilutions of macerated tissue generated from the incubation of potato tubers with either the wild-type or the *rsmA^−^* strain were plated on LB. After 20 h, in experiments with both strains the total bacterial numbers reached ∼10^9^ CFU per gram of macerated tissue (Fig. 10B). The plates with the wild-type strain contained uniform colonies with growth rate and PCWDE production identical to the original *Pw* strain. However, the LB plates inoculated with the dilutions of the *rsmA^−^* macerated tissue contained colonies with variable colony morphologies and growth rates. This prompted us to plate the *rsmA^−^* macerated tissue in parallel to the selective LB+Cm plates. The number of cells growing in the presence of Cm was 2 magnitudes lower than the total colony count. Therefore, it appears that the *rsmA^−^* strain represents only a minority of the bacteria present in macerated tissue. Moreover, the bacterial count in control tubers, inoculated with the buffer not generating macerated tissue was quite high, revealing the presence of resident microflora in the tubers (Fig. 10B). After 40 h of incubation the *rsmA^−^* cells were almost completely disappeared and the infection site was taken over by the indigenous bacteria although the amount of macerated tissue was high. Taken together, these results demonstrate that although the *rsmA^−^* mutant is able to produce more virulence factors and causes more damage to plant tissue, its ability to cope with the resident micro flora or other conditions found in the potato tubers is reduced compared to the wild-type strain.

The effects of *rsmA^−^* mutation on cell and flagella morphology were also examined in macerated tissues. At both time points, 20 h and 40 h, wild-type cells were observed to be elongated and hyperflagellated. However, for the *rsmA^−^* cells at the 20 h time point, cells were extremely long, filamentous, thin, and hyperflagellated, similar to the phenotype observed for *rsmA^−^* cells on swarming plates. After 40 h, it was difficult to detect *rsmA^−^* cells due to the presence of numerous types of bacteria with various cell shapes.

In conclusion, the similarity of wild-type cells swarming on agar plates containing potato extract and cells isolated from potato tubers following infection suggests that the physiological state of potato-infecting bacteria is similar to that of the swarming state. Moreover, the increase in swarming activity observed for the *rsmA^−^* mutant suggests that RsmA is involved in the switch to a swarming phenotype.

## Discussion

The microarray data collected in this study reveal global changes in physiology and metabolism occur in *Pw* with the loss of *rsmA*. Some of these changes involve the synthesis of PCWDE and other virulence factors, flagella synthesis and motility, the switch from glycogen production to glycogen consumption, increased citrate uptake, butanediol fermentation, and regulation of the TCA cycle to maximum energy production. These physiological processes are required for adaptation at the site of bacterial invasion in response to a host plant. In addition, when microarray data from deletion of *rsmA* were compared with *rsmA* overexpression in *Pa* (i.e., the *expI^−^* mutant) [3], several gene clusters strongly repressed by RsmA were identified. In particular, type VI secretion and phosphonate metabolism. The expression of these gene clusters has been shown to be promoted by plant extracts [47], although their functions in relation to virulence are unknown. We identified many additional genes that are oppositely regulated in response to *rsmA* deletion and overexpression. As these genes have no obvious role in virulence, these were not analyzed in the current study and await for future investigations.

The slow growth phenotype of *rsmA^−^* cells could be considered similar to those of strains expressing large quantities of non-functional, non-toxic proteins from multi-copy plasmids. In the case of *rsmA^−^* cells, the “gratuitous” proteins are the highly produced PCWDEs, and these are not essential for bacterial growth. It has been shown that the accumulation of excessive proteins is accompanied by a progressive decrease in cell growth rates [48]. High growth rates require the continuous production of housekeeping proteins. Due to limited quantities of free RNA polymerase and ribosomes in the cell, the expression of excessive proteins thereby leads to a decrease in growth rate [49].

In the *rsmA* knock out mRNA levels of genes involved in the glyoxylate shunt were found to be down-regulated, and genes for the TCA cycle were up-regulated (Figs. 2, 7). The glyoxylate bypass can provide anabolic intermediates; the TCA cycle is more efficient at providing energy. The glyoxylate shunt enzyme, isocitrate lyase (AceA) competes with the TCA cycle enzyme, isocitrate dehydrogenase (the product of *icd*), for isocitrate. While isocitrate dehydrogenase has a much higher affinity for isocitrate, it is inactivated by phosphorylation when ATP concentrations are high and the cell needs precursors for biosynthesis [50]. Inhibition of isocitrate dehydrogenase then slows the TCA cycle and forces isocitrate through the bypass. When metabolic intermediates and AMP/ADP accumulate, isocitrate dehydrogenase is then dephosphorylated to function in the TCA cycle [50]. In the *rsmA^−^* mutant, increased mRNA levels of genes involved in TCA cycle might be a response to the limited energy resources of these cells. However, whether the up-regulation of proteins in the TCA cycle and the down-regulation of proteins that are part of the gyoxylate shunt is a consequence of the higher energy demand of the *rsmA^−^* strain, or is the direct result of gene regulation by RsmA, remains unsolved.

Both wild-type and *rsmA^−^ Pw* cells exhibited a swarming phenotype in potato tubers. In animal pathogens such as *Proteus mirabilis*, *Salmonella typhimurium*, *Serratia marcescens*, *Pseudomonas aeruginosa*, and *Bacillus cereus*, the swarming process is coupled with the invasion of new host cells and virulence factor synthesis [51]–[54]. Furthermore, transcriptome studies of these bacteria have indicated that profound differences exist between swimmer and swarmer cells [51]. For example, morphological changes of swarmer cells include an increase in the number of flagella and cell elongation. Swarmer cell differentiation results in substantial alterations in metabolism and environmental survival strategies. Regarding the latter, these changes indicate that swarming represents a complex lifestyle adaptation. It has also been demonstrated in *Salmonella* and *E. coli* that a full TCA cycle is needed for swarming motility, a behavior that consumes a lot of energy [51],[55]. Similarly, up-regulation of the TCA cycle in the *Pw rsmA^−^* strain would be predicted to be needed for up-regulation of virulence factor synthesis.

It is commonly believed that swarming cells suppress cell division, and that cell elongation is either a requirement for, or an indicator of, swarming motility. Regarding the filamentous cell shape of *Pw* swarming cells, there are three possible explanations. First, due to the energy required for cells to undergo swarming, cell division could be inhibited, thereby resulting in a filamentous cell shape. Secondly, stress induced by an antibiotic-like compound could be a factor. For example, swarming stimuli are poorly understood. However, they are hypothesized to be induced by environmental signals [56]. In *Pw*, elongated cell shapes and hyperflagellation are promoted by a host. In this study, potato tubers or potato tuber extract were used to represent a host. If certain plant components act as an antibiotic, cell filamentation could be induced. Moreover, in potato tubers, the more susceptible *rsmA^−^* strain was observed to undergo cell death. Thirdly, it is possible that the filamentous cell shape is induced by a potato tuber component which does not cause stress, but rather improves movement in the host.

The most probable reason for the low cell number observed for the *rsmA ^−^*strain in potato tubers is unregulated virulence factor synthesis. For example, once a pathogen penetrates a plant cellulose-based cell wall, the pathogen is exposed to plant extracellular surface receptors that recognize pathogen-associated molecular patterns (PAMP) such as bacterial flagellin and EF-Tu [57], [58]. This recognition triggers immunity, which usually halts an infection before a microbe can establish itself in the plant [59], [60]. Therefore, during infection, pathogens actively suppress a plant's PAMP-triggered defenses [61]. However, plants respond to cell wall damage by activating a variety of defenses [62]. In the *rsmA^−^* strain, the Rsm system is compromised, thereby disrupting induction by quorum sensing and several other regulatory pathways. As a result, deregulated synthesis of PCWDE, flagellin, and other virulence factors occurs. Production of these proteins occurs in the *rsmA* ^−^ mutant at higher level than in wild-type and may start before the bacteria have reached the critical cell concentration for attacking the plant. Flagellin, EF-Tu, acetoine, butanediol and a damaged cell wall can trigger plant defense responses [43], [44], [57], [58], which a single bacterial cell, although well equipped, cannot resist.

When studying bacteria numbers in infected potato tubers, it was found that despite surface-sterilization with hypochlorite acid prior to inoculation, the resident bacterial community of potato tubers can survive in considerable numbers. This may be due to fissures in the potato peel or bacteria present within the potato. In *rsmA^−^* generated lesions, the number of bacteria present was particularly high, and included a variety of colony structures and sizes. This may be due to increased expression of PCWDE in the *rsmA* ^−^ strain that could lead to the release of plant components at high levels. However, bacteria intrinsic to the potatoes used for inoculation were also present for the wild-type experiments, indicating that the wild-type *Pw* strain was able to efficiently out-compete these organisms. We hypothesize that the maceration of potato tubers is achieved by a diverse population of bacteria. For example, *Pw* cannot degrade starch, which is the main carbohydrate present in potato tubers. Degradation of the plant cell wall by PCWDE of *Pw* could expose the starch present to other bacteria.

In conclusion, plant invasion is a hazardous and energy consuming process for bacteria, and accordingly, has to be tightly regulated. Therefore, the unregulated production of virulence factors at high levels in the *rsmA^−^* strain is energetically costly, and can reduce the ability of *Pw* to invade a plant and compete with the natural bacterial community of the host. Moreover, in *Pw*, an attack of a plant and the induction of virulence factors are accompanied by a swarming phenotype.

## Supporting Information

Figure S1 The Rsm system affects the ability of *Pw* to infect tobacco seedlings. Surface inoculations were performed on 3-to-4-week-old seedlings, with 10^8^ wild-type, *rsmA*-, or *rsmB*-defective bacteria applied per seedling. A total of 24 seedlings were inoculated for each strain. White and black bars indicate the percentage of tobacco seedlings macerated 24 or 72 h post-inoculation, respectively.
(TIF)

Figure S2 Expression of a *prtW* reporter gene fusion is dependent on the Rsm system. A *prtW::gusA* fusion was used to evaluate the effect of either *rsmB* or *rsmA* inactivation on protease (PrtW) expression. Cells were grown in minimal medium supplemented with 10% celery extract, and *β*-glucuronidase (GusA) activity was assayed in the exponential phase (white) and the early stationary phase (black) for each strain (*i.e.*, 6 and 10 h post inoculation for the wild-type and *rsmB^−^* strains; and 10 and 18 h post inoculation for the *rsmA* ^−^ strain). The experiment was performed in triplicate and error bars indicate standard deviation values.
(TIF)
