# Correction: Lack of RsmA-Mediated Control Results in Constant Hypervirulence, Cell Elongation, and Hyperflagellation in *Pectobacterium wasabiae*

**DOI:** 10.1371/annotation/6bc0b20b-3736-431f-9687-67986101128f

**Published:** 2013-12-16

**Authors:** Viia Kõiv, Liis Andresen, Martin Broberg, Jekaterina Frolova, Panu Somervuo, Petri Auvinen, Minna Pirhonen, Tanel Tenson, Andres Mäe

The author list for the article should be corrected to include E. Tapio Palva. The revised author list should read as follows:

Viia Kõiv, Liis Andresen, Martin Broberg, Jekaterina Frolova, Panu Somervuo, Petri Auvinen, E. Tapio Palva, Minna Pirhonen, Tanel Tenson, Andres Mäe

Dr. Palva provided the DNA sequence information of Pectobacterium wasabiae SCC3193, and contributed to the microarray analyses for the study and to the analysis of the results. The details of authors' contributions should also be corrected to reflect the following contributions:

Conceived and designed the experiments: VK LA ETP. Performed the experiments: VK LA MB JF ETP. Analyzed the data: VK LA MB ETP. Contributed reagents/materials/analysis tools: PS PA MP TT AM. Wrote the paper: VK LA MP TT AM. 

