# Supplementary figures and images for: Lack of RsmA-Mediated Control Results in Constant Hypervirulence, Cell Elongation, and Hyperflagellation in Pectobacterium wasabiae
Source: PLoS One. 2013 Jan 23;8(1):e54248. doi: 10.1371/journal.pone.0054248 (PMC3553148; doi:10.1371/journal.pone.0054248)

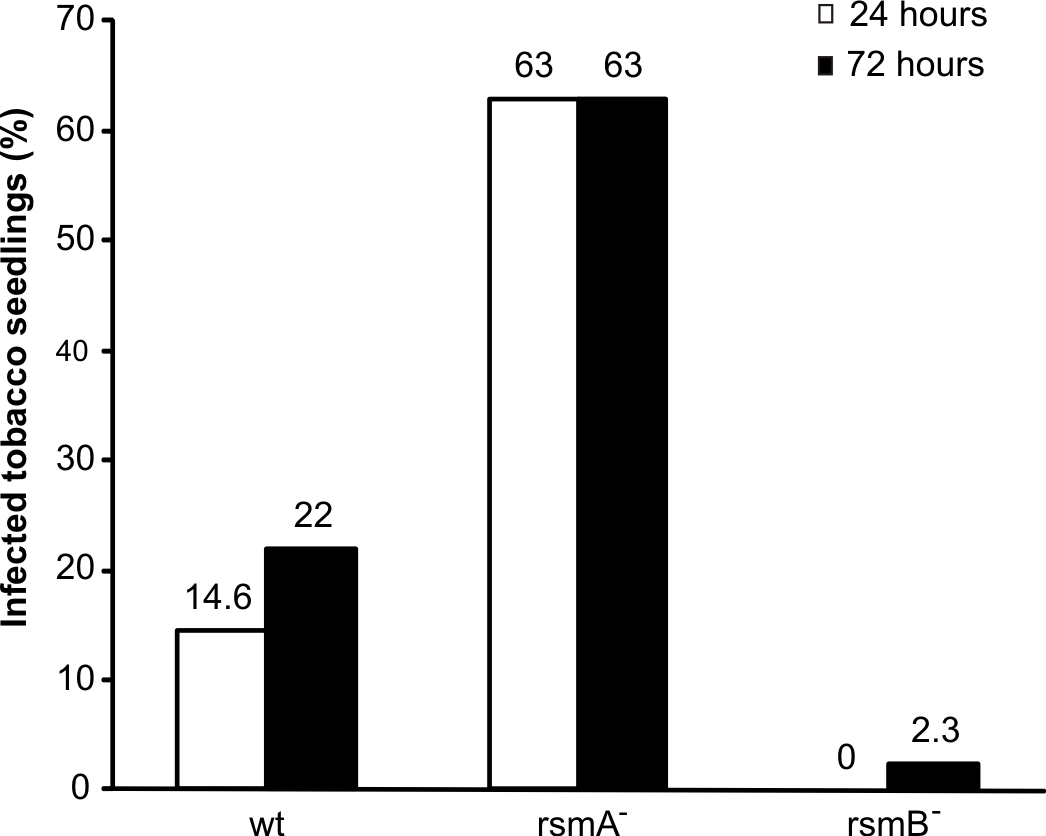

Supplement: Figure S1 — The Rsm system affects the ability of Pw to infect tobacco seedlings. Surface inoculations were performed on 3-to-4-week-old seedlings, with 108 wild-type, rsmA-, or rsmB-defective bacteria applied per seedling. A total of 24 seedlings were inoculated for each strain. White and black bars indicate the percentage of tobacco seedlings macerated 24 or 72 h post-inoculation, respectively. (TIF) [file pone.0054248.s001.tif]

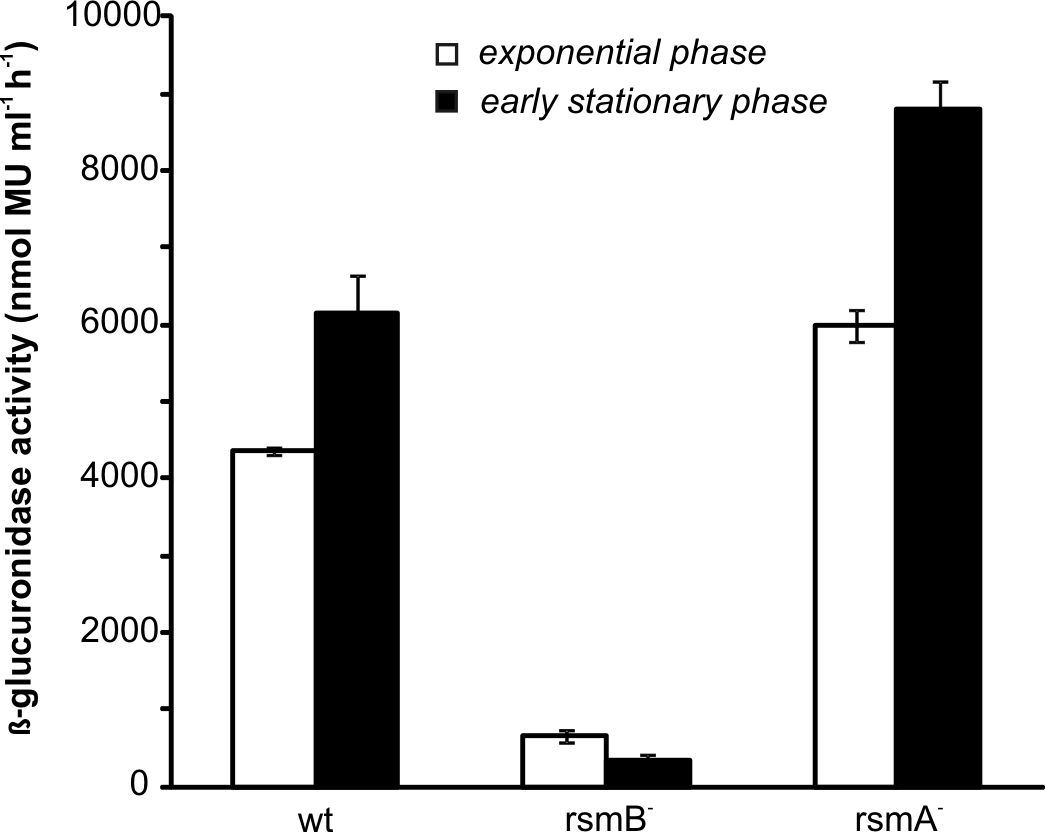

Supplement: Figure S2 — Expression of a prtW reporter gene fusion is dependent on the Rsm system. A prtW::gusA fusion was used to evaluate the effect of either rsmB or rsmA inactivation on protease (PrtW) expression. Cells were grown in minimal medium supplemented with 10% celery extract, and β-glucuronidase (GusA) activity was assayed in the exponential phase (white) and the early stationary phase (black) for each strain (i.e., 6 and 10 h post inoculation for the wild-type and rsmB− strains; and 10 and 18 h post inoculation for the rsmA − strain). The experiment was performed in triplicate and error bars indicate standard deviation values. (TIF) [file pone.0054248.s002.tif]
